# Supplementary material for: Dielectric Mie voids: confining light in air
Source: Light Sci Appl. 2023 Jan 1;12:3. doi: 10.1038/s41377-022-01015-z (PMC9805462; doi:10.1038/s41377-022-01015-z)
Supplement: Supplementary file 1 — Supporting Information [file 41377_2022_1015_MOESM1_ESM.docx]

**Supporting Information**

**for**

**“Dielectric Mie Voids: Confining Light in Air”**

Mario Hentschel^1,*^, Kirill Koshelev^2^, Florian Sterl^1^, Steffen Both^1^, Julian Karst^1^, Lida Shamsafar^1^, Thomas Weiss^1,3^, Yuri Kivshar^2,*^, and Harald Giessen^1,*^

*^1^4th Physics Institute and Research Center SCoPE, University of Stuttgart, Pfaffenwaldring 57, 70569 Stuttgart, Germany*

*^2^Nonlinear Physics Centre, Research School of Physics, Australian National University, Canberra ACT 2601, Australia*

*^3^Institute of Physics, University of Graz, and NAWI Graz, Universitätsplatz 5, 8010 Graz, Austria*

^*^m.hentschel@physik.uni-stuttgart.de, yuri.kivshar@anu.edu.au, giessen@physik.uni-stuttgart.de


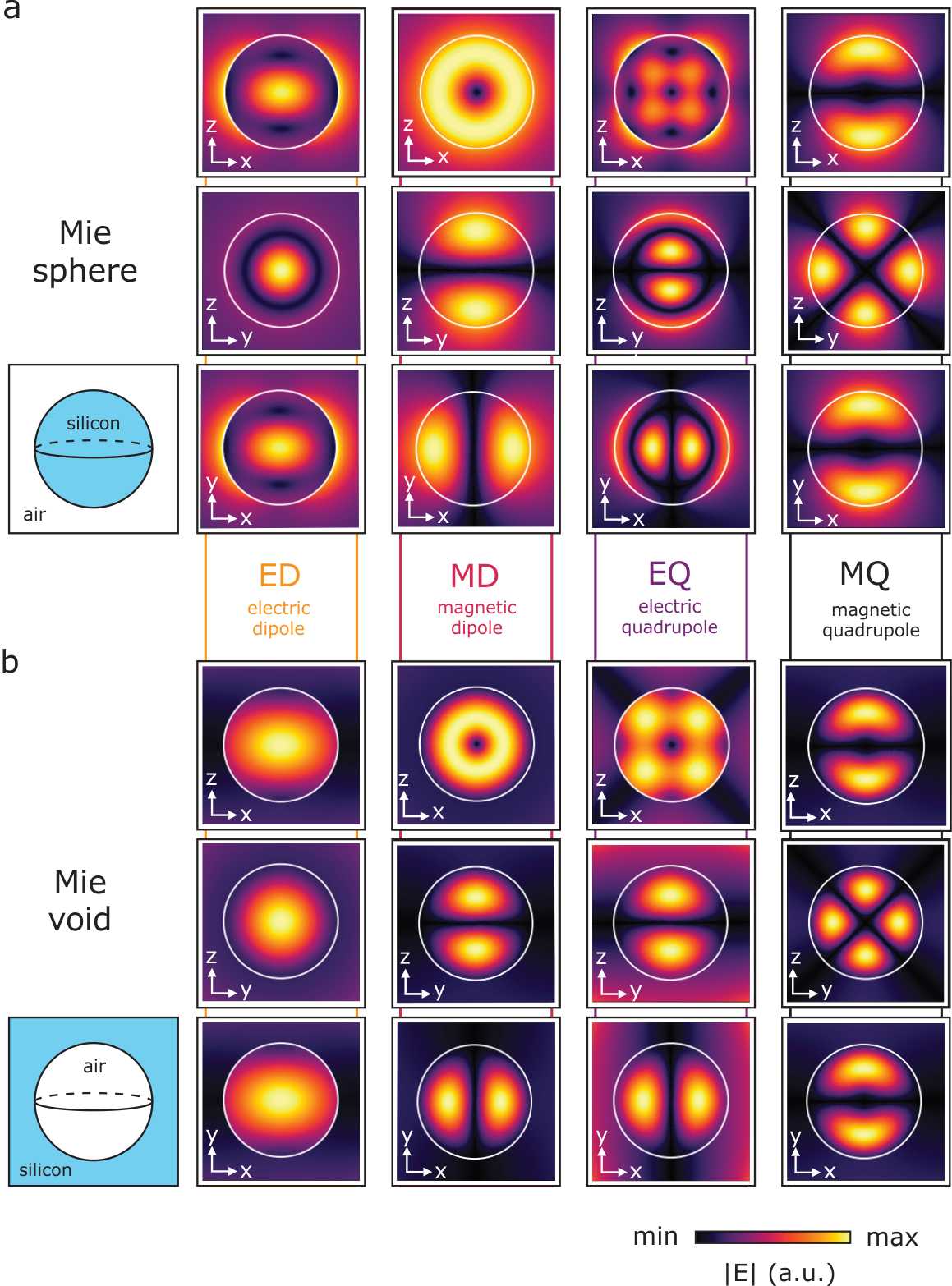


**Figure S1**: Electric field profiles for the eigenmodes of (a) a silicon sphere in air and (b) a spherical air void in silicon environment. The fields are for four fundamental modes, electric and magnetic dipole, and electric and magnetic quadrupole. The field cross-sections are plotted in the main X-Z, Y-Z, and X-Y planes for the sphere centered in the origin of the reference frame. Each of the selected modes is degenerate with respect to the azimuthal index *m*, so the field profiles are shown only for the modes that can be excited with a linearly X-polarized plane wave propagating along the Z direction similar to conditions of excitation in the experiment (Fig. 3 of the main text). The selected modes correspond to the azimuthal index *m* = -1 for magnetic modes and *m* = 1 for electric modes. The refractive index of silicon in simulations is 3.7, the extinction coefficient is zero.

**
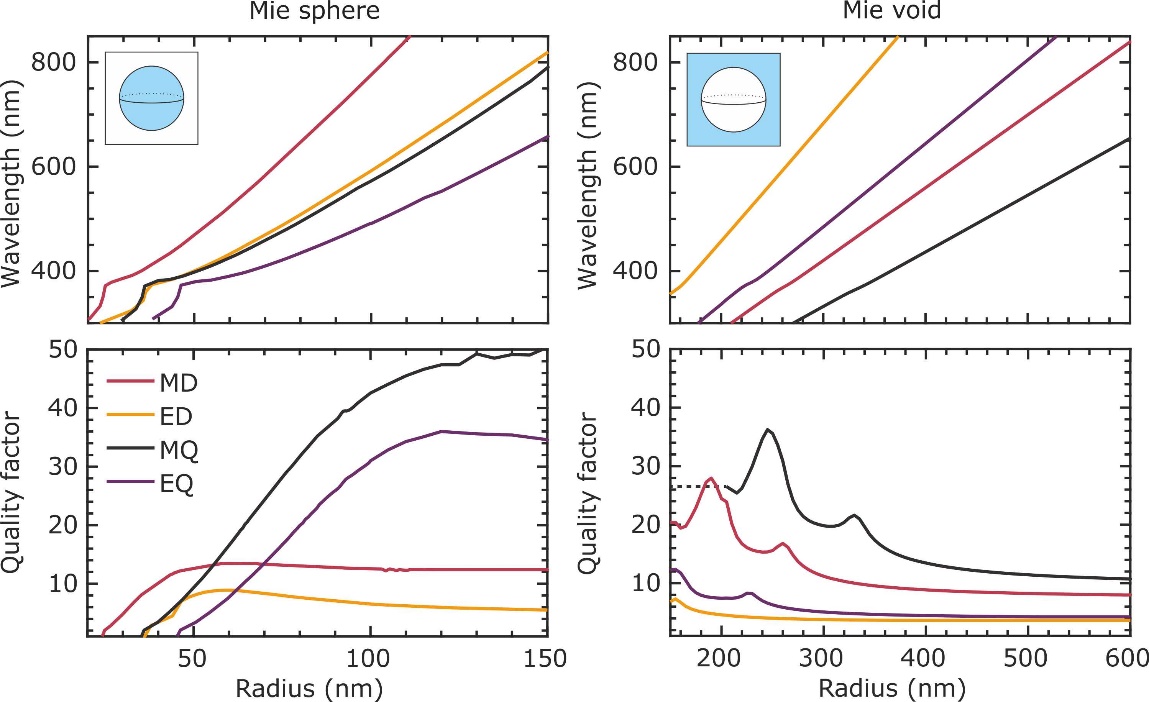
**

**Figure S2**: Calculated dependence of the resonant wavelength and mode quality factor on the sphere radius for a silicon sphere in air (left panels) and a spherical air void in silicon environment (right panels). The refractive index and extinction coefficients of silicon are taken from Fig.2c in the main text (top panel). The calculations are done for four fundamental modes, electric and magnetic dipole, and electric and magnetic quadrupole. The dashed line in lower right panel shows the extrapolation of quality factor to the size range where the dispersion of material is unavailable (wavelength range below 290 nm). The sharp peaks of the quality factor for Mie voids for sphere radius below 350 nm can be explained by increase of the extinction coefficient and refractive index, which leads to increased Fresnel reflection coefficient from the air-silicon interface.

**
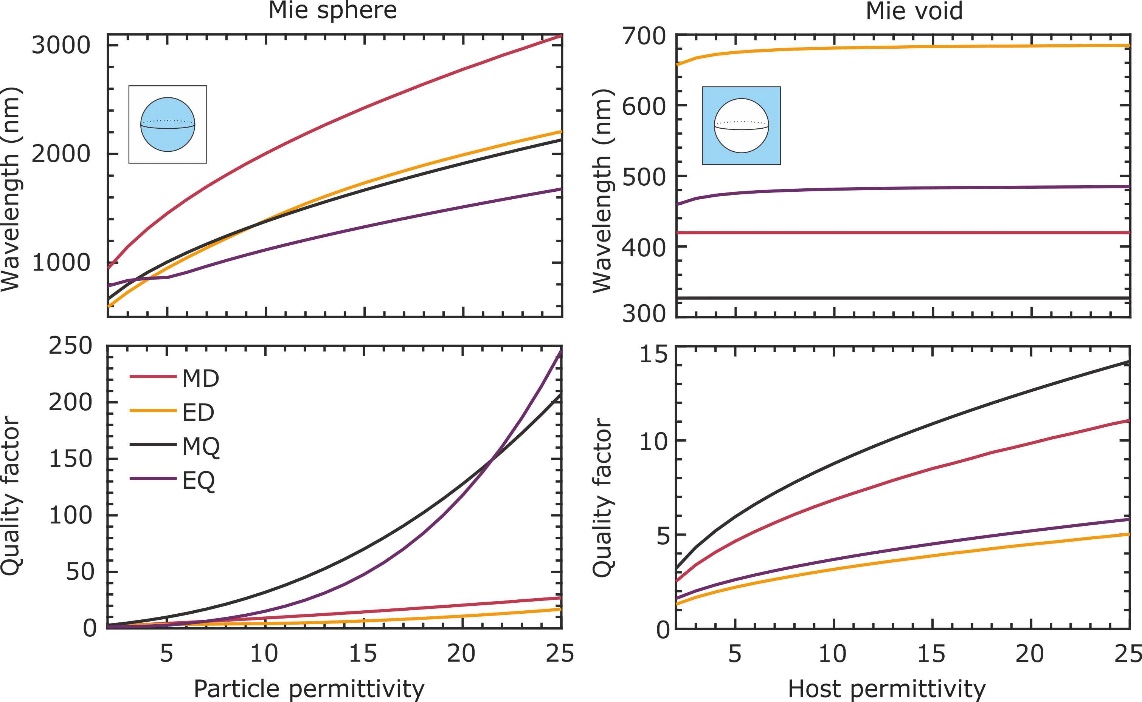
**

**Figure S3**: Calculated dependence of the resonant wavelength and mode quality factor on the material permittivity for a dielectric sphere in air (left panels) and a spherical air void in dielectric environment (right panels). The dielectric material is lossless. The calculations are done for four fundamental modes, electric and magnetic dipole, and electric and magnetic quadrupole. While the modes of the dielectric sphere show strong dispersion for varying permittivity values of the dielectric, the resonant wavelength of modes of a Mie void is robust to the change of host permittivity as the modes are confined in air. The quality factors of the modes for the dielectric sphere exhibit an extremely strong dependence on the permittivity and increase significantly for increasing permittivity. A similar behaviour is observed for the void, yet, the overall change is weaker. Again, the difference can be explained by the confinement in the dielectric vs. confinement in air.


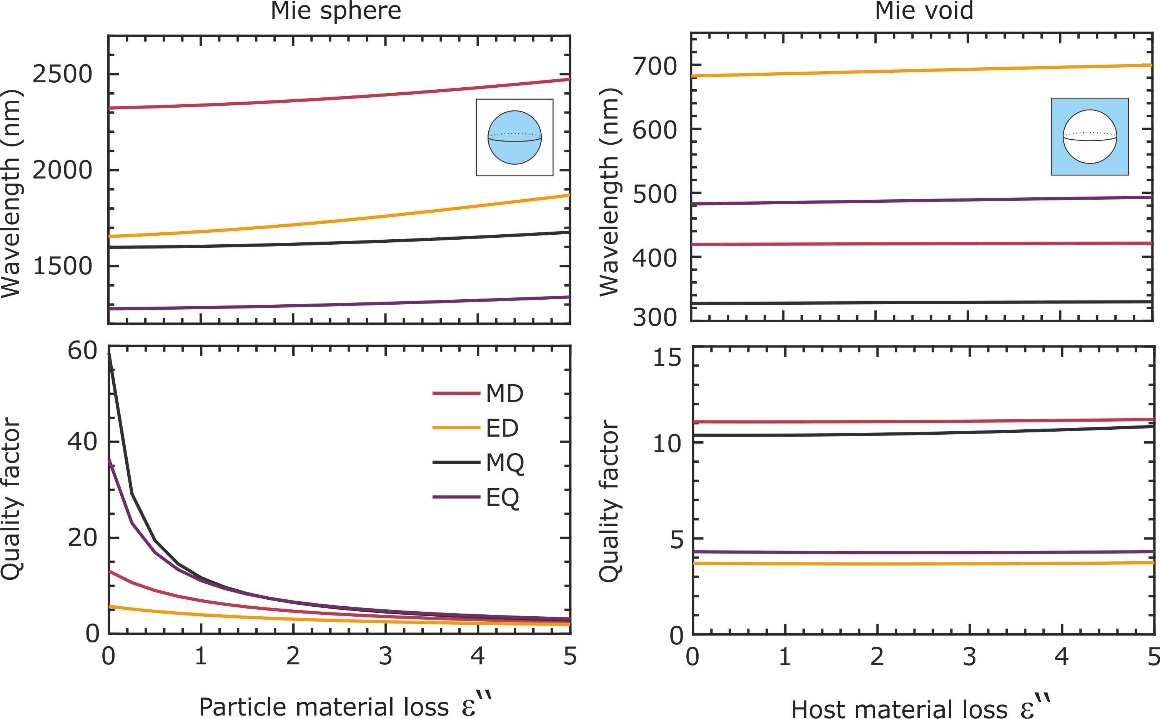


**Figure S4**: Calculated dependence of the resonant wavelength and mode quality factor on material losses for a dielectric sphere in air (left panels) and a spherical air void in dielectric environment (right panels). The real part of the dielectric’s permittivity is 13.69. The calculations are done for four fundamental modes, electric and magnetic dipole, and electric and magnetic quadrupole. The resonant wavelength of the modes for the dielectric sphere exhibit a slow increase with increasing loss while the quality factor drops extremely fast with increasing loss. Both phenomena are explained by the confinement of the mode to the lossy dielectric material and thus are subject to strong damping. The resonant wavelength of modes of a Mie void is robust to the increase of losses, while their quality factor grows slowly with increase of material absorption. Due to the confinement of the mode to air the losses of the host material do not damp the mode. The slight increase in the quality factor can be explained by an increased reflection at the air/silicon interface with increasing loss and thus by a stronger confinement.


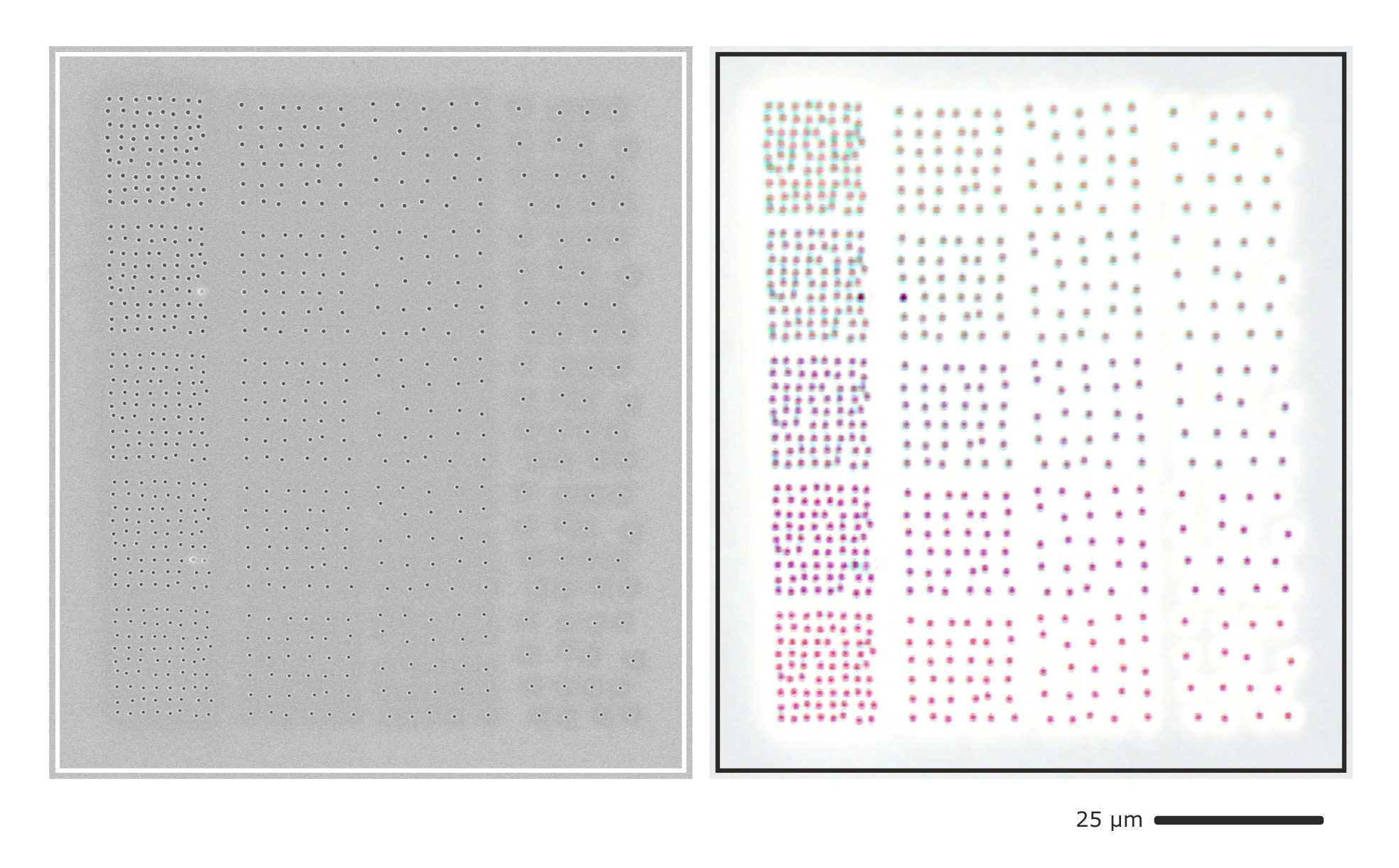


**Figure S5**: Disordered gratings of periods 2 µm, 3 µm, 4 µm, and 5 µm with varying particle diameter (bottom to top). All rows show distinctively different colours that remain unchanged with increasing periodicity, showing that the colour impression is indeed caused by localized modes. Also note the structuring defects in the second and fourth row, first column, which can be clearly identified in the colour impression, also pointing to localized modes of each particle.


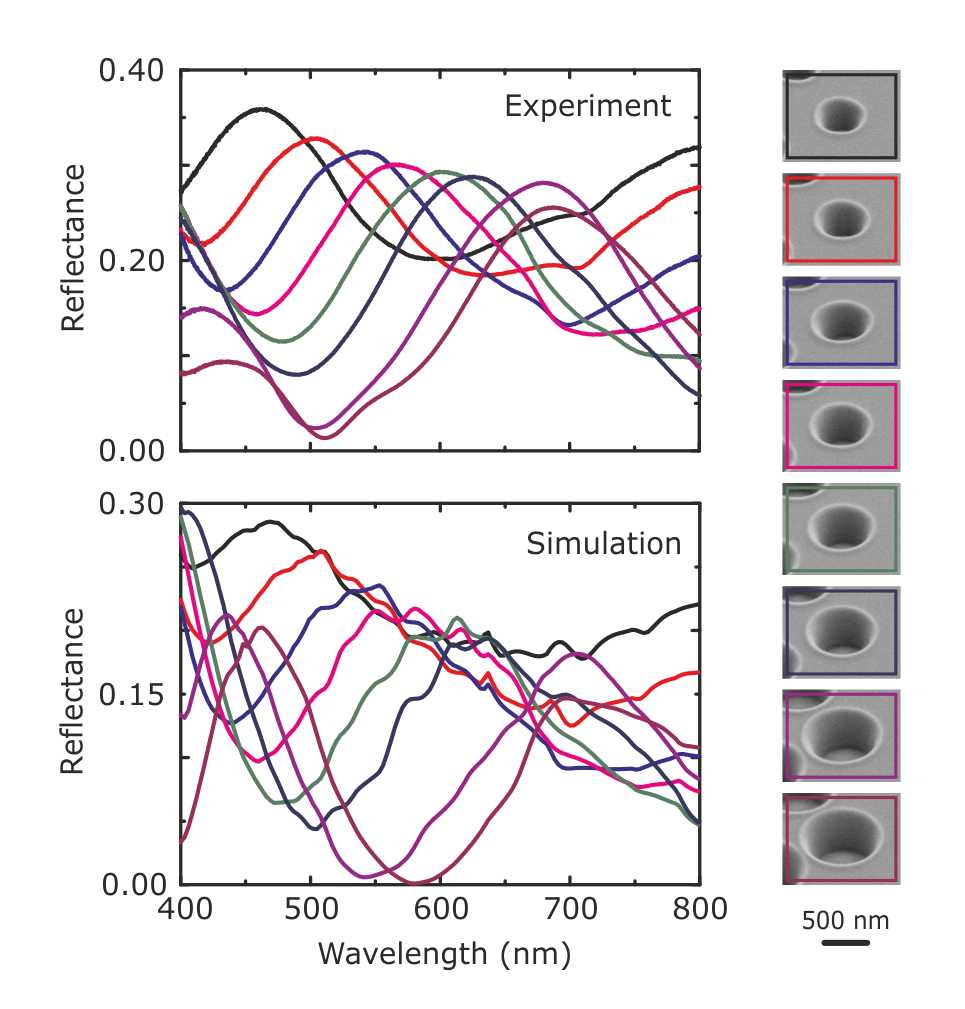


**Figure S6**: Experimental and simulated reflectance spectra as well as SEM images of the structures shown in Figure 3 in the main text (without vertical displacement). The experimentally determined geometrical parameters are as follows: (upper radius (nm), lower radius (nm), depth (nm)): (225, 135, 400), (255, 145, 400), (290, 160, 400), (305, 185, 410), (330, 200, 420), (340, 225, 440), (390, 265, 470), (405, 290, 500). These values are also used for the simulation. Periodicity is 900 nm in both directions.

**
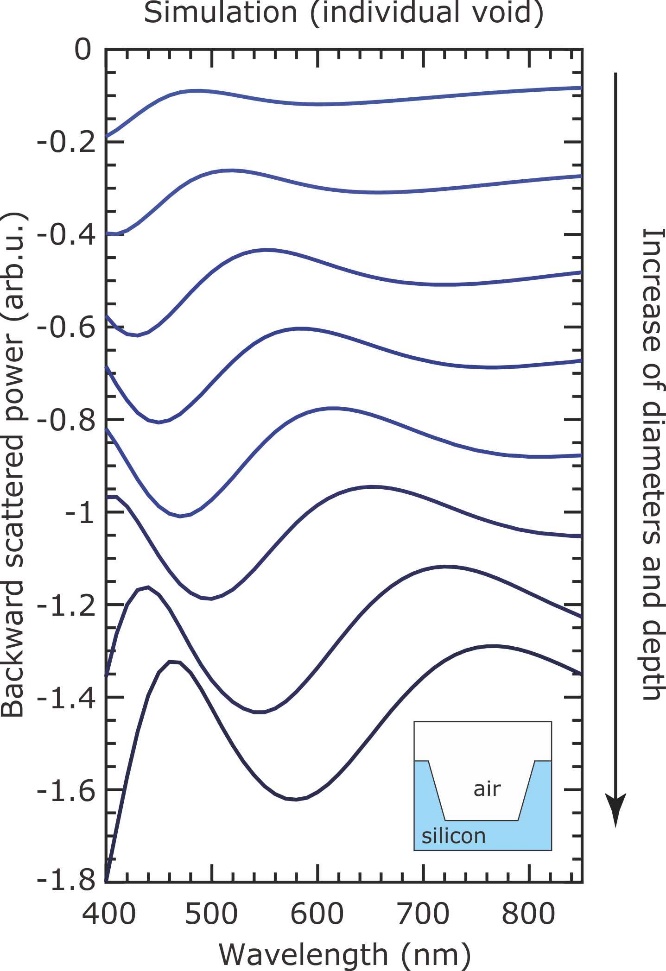
**

**Figure S7**: Simulated spectra of backward-scattered power from a single air void in silicon substrate for different diameters and depths. The geometry of the void and the range of size parameters are the same as in Fig. 3c of the main text. The resulting power is calculated as the difference between the backward-scattered power from the silicon substrate with a void and a bare silicon substrate. The refractive index and extinction coefficients of silicon are taken from the data shown in Fig. 2c of the main text (top panel). The spectra are shifted vertically relatively by 0.17 units. We observe excellent agreement with the measurement and finite-element-based array simulations shown in Fig. 3b. This behaviour underpins the localized nature of the observed modes and the minute to vanishing contribution from array or grating effects.

**
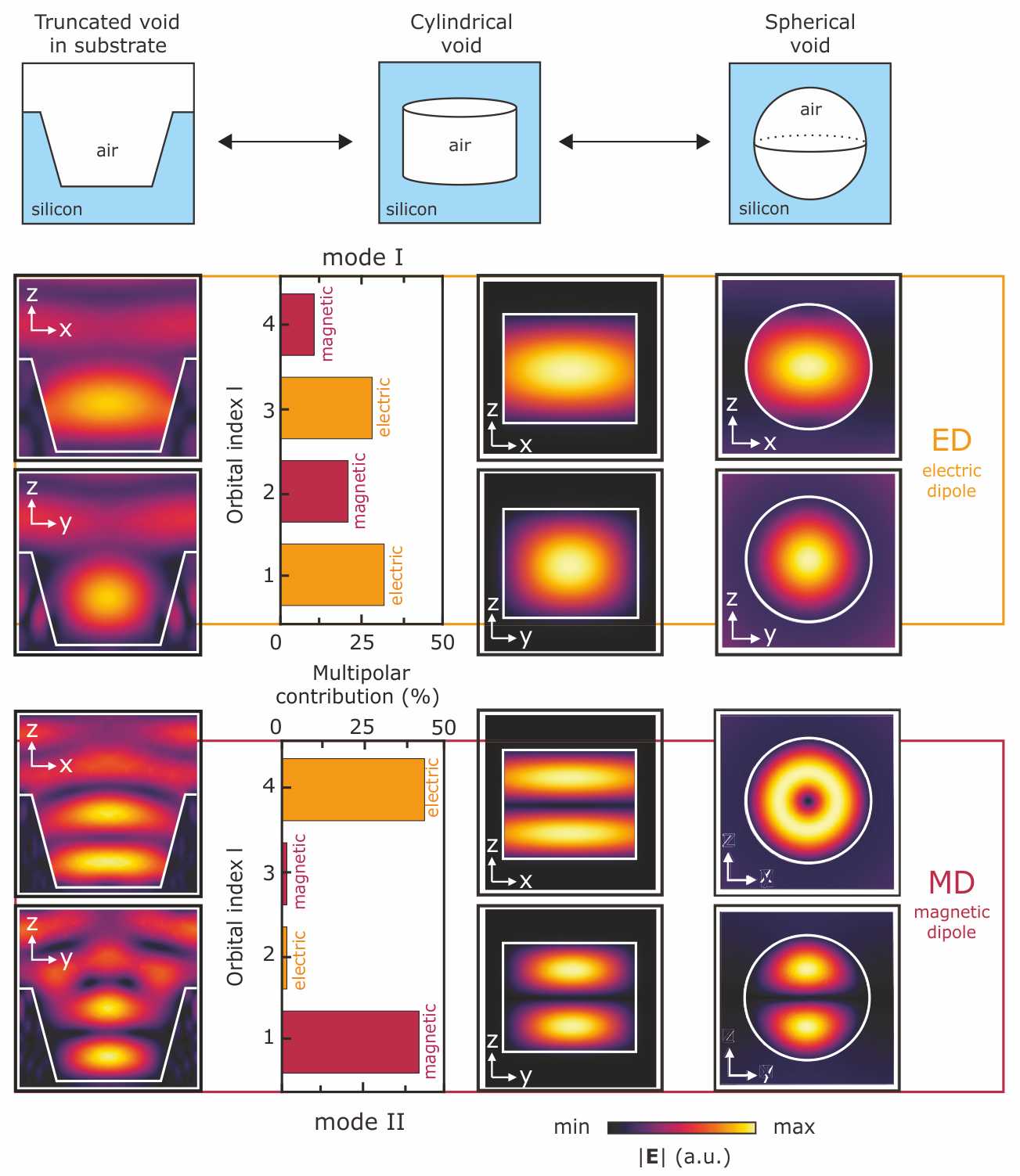
**

**Figure S8**: Connection between modes I and II of a cylindrical void in substrate and modes of a spherical Mie void. Comparison of the electric field profiles in Z-X and Z-Y planes for ED and MD modes of a spherical air void in homogeneous silicon environment (right) with the modes I and II, respectively, for a cylindrical air void in homogeneous silicon environment (central), and a truncated cylindrical air void in a silicon substrate (left). The field profiles for the central and right columns are from the eigenmode simulations, and for the left column are from the scattering simulations for periodic structures as in Fig. 3c of the main text. The inset on the top shows the geometry of the corresponding column. Overall, we observe a very good agreement between the mode profiles for cylindrical-shaped geometries (left and central) obtained from the full-wave reflection simulation and the eigenmode analysis. Moreover, in comparing these modal profiles to the analytical modes obtained from Mie’s theory on the right, we can identify the key properties of the electric and magnetic dipolar modes in the simulated ones. This is particularly clear for the electric dipolar mode, for which we observe excellent match. To further quantify this, the insets on the left show the relative multipolar contribution of the first four fundamental multipolar channels with the orbital indices from 1 to 4 to the radiated field of modes I and II for the cylindrical air void in homogeneous silicon environment. The comparison of the field profiles and multipolar decomposition results show that mode I is dominated by the electric dipolar radiation and mode II is dominated by the magnetic dipolar radiation with a contribution from other channels.

|  | 1 | 2 | 3 | 4 | 5 | 6 | 7 | 8 | 9 | 10 | 11 | 12 | 13 | 14 | 15 | 16 | 17 | 18 | 19 | 20 | 21 | 22 | 23 | 24 | 25 | 26 | 27 | 28 | 29 | 30 | 31 | 32 | 33 | 34 | 35 | 36 |
| --- | --- | --- | --- | --- | --- | --- | --- | --- | --- | --- | --- | --- | --- | --- | --- | --- | --- | --- | --- | --- | --- | --- | --- | --- | --- | --- | --- | --- | --- | --- | --- | --- | --- | --- | --- | --- |
| 36 | 337 | 360 | 383 | 406 | 429 | 465 | 500 | 536 | 571 | 607 | 621 | 635 | 649 | 663 | 677 | 698 | 719 | 740 | 761 | 782 | 800 | 818 | 837 | 855 | 873 | 891 | 909 | 927 | 945 | 963 | 982 | 1000 | 1018 | 1036 | 1054 | 1072 |
| 35 | 328 | 350 | 373 | 395 | 418 | 452 | 487 | 521 | 556 | 590 | 604 | 618 | 631 | 645 | 659 | 679 | 700 | 720 | 741 | 761 | 779 | 796 | 814 | 832 | 849 | 867 | 885 | 902 | 920 | 937 | 955 | 973 | 990 | 1008 | 1026 | 1043 |
| 34 | 319 | 341 | 363 | 384 | 406 | 440 | 473 | 507 | 540 | 574 | 587 | 601 | 614 | 627 | 641 | 660 | 680 | 700 | 720 | 740 | 757 | 774 | 791 | 809 | 826 | 843 | 860 | 877 | 894 | 911 | 929 | 946 | 963 | 980 | 997 | 1014 |
| 33 | 310 | 331 | 352 | 373 | 394 | 427 | 460 | 492 | 525 | 558 | 571 | 583 | 596 | 609 | 622 | 642 | 661 | 680 | 699 | 719 | 735 | 752 | 769 | 785 | 802 | 819 | 835 | 852 | 869 | 885 | 902 | 919 | 935 | 952 | 969 | 985 |
| 32 | 301 | 321 | 342 | 363 | 383 | 414 | 446 | 478 | 510 | 541 | 554 | 566 | 579 | 591 | 604 | 623 | 641 | 660 | 679 | 698 | 714 | 730 | 746 | 762 | 778 | 795 | 811 | 827 | 843 | 859 | 875 | 892 | 908 | 924 | 940 | 956 |
| 31 | 292 | 312 | 332 | 352 | 371 | 402 | 433 | 463 | 494 | 525 | 537 | 549 | 561 | 574 | 586 | 604 | 622 | 640 | 658 | 676 | 692 | 708 | 724 | 739 | 755 | 771 | 786 | 802 | 818 | 833 | 849 | 865 | 880 | 896 | 912 | 927 |
| 30 | 282 | 302 | 321 | 341 | 360 | 389 | 419 | 449 | 479 | 508 | 520 | 532 | 544 | 556 | 567 | 585 | 603 | 620 | 638 | 655 | 671 | 686 | 701 | 716 | 731 | 746 | 762 | 777 | 792 | 807 | 822 | 838 | 853 | 868 | 883 | 898 |
| 29 | 273 | 292 | 311 | 330 | 348 | 377 | 406 | 434 | 463 | 492 | 503 | 515 | 526 | 538 | 549 | 566 | 583 | 600 | 617 | 634 | 649 | 664 | 678 | 693 | 708 | 722 | 737 | 752 | 767 | 781 | 796 | 811 | 825 | 840 | 855 | 869 |
| 28 | 264 | 282 | 300 | 319 | 336 | 364 | 392 | 420 | 448 | 476 | 487 | 498 | 509 | 520 | 531 | 547 | 564 | 580 | 597 | 613 | 627 | 641 | 656 | 670 | 684 | 698 | 713 | 727 | 741 | 755 | 769 | 784 | 798 | 812 | 826 | 840 |
| 27 | 255 | 273 | 290 | 308 | 325 | 352 | 379 | 405 | 432 | 459 | 470 | 481 | 491 | 502 | 512 | 528 | 544 | 560 | 576 | 592 | 606 | 619 | 633 | 647 | 661 | 674 | 688 | 702 | 715 | 729 | 743 | 757 | 770 | 784 | 798 | 811 |
| 26 | 246 | 263 | 280 | 297 | 313 | 339 | 365 | 391 | 417 | 443 | 453 | 463 | 474 | 484 | 494 | 509 | 525 | 540 | 555 | 571 | 584 | 597 | 610 | 624 | 637 | 650 | 663 | 677 | 690 | 703 | 716 | 730 | 743 | 756 | 769 | 782 |
| 25 | 237 | 253 | 269 | 286 | 302 | 327 | 352 | 376 | 401 | 426 | 436 | 446 | 456 | 466 | 476 | 491 | 505 | 520 | 535 | 550 | 562 | 575 | 588 | 601 | 613 | 626 | 639 | 652 | 664 | 677 | 690 | 703 | 715 | 728 | 741 | 753 |
| 24 | 228 | 243 | 259 | 275 | 290 | 314 | 338 | 362 | 386 | 410 | 420 | 429 | 439 | 448 | 458 | 472 | 486 | 500 | 514 | 529 | 541 | 553 | 565 | 578 | 590 | 602 | 614 | 627 | 639 | 651 | 663 | 676 | 688 | 700 | 712 | 725 |
| 23 | 219 | 234 | 249 | 264 | 278 | 301 | 324 | 348 | 371 | 394 | 403 | 412 | 421 | 430 | 439 | 453 | 467 | 480 | 494 | 507 | 519 | 531 | 543 | 554 | 566 | 578 | 590 | 601 | 613 | 625 | 637 | 648 | 660 | 672 | 684 | 696 |
| 22 | 210 | 224 | 238 | 253 | 267 | 289 | 311 | 333 | 355 | 377 | 386 | 395 | 403 | 412 | 421 | 434 | 447 | 460 | 473 | 486 | 497 | 509 | 520 | 531 | 543 | 554 | 565 | 576 | 588 | 599 | 610 | 621 | 633 | 644 | 655 | 667 |
| 21 | 200 | 214 | 228 | 242 | 255 | 276 | 297 | 319 | 340 | 361 | 369 | 378 | 386 | 394 | 403 | 415 | 428 | 440 | 453 | 465 | 476 | 487 | 497 | 508 | 519 | 530 | 541 | 551 | 562 | 573 | 584 | 594 | 605 | 616 | 627 | 638 |
| 20 | 191 | 204 | 218 | 231 | 244 | 264 | 284 | 304 | 324 | 344 | 352 | 360 | 368 | 376 | 384 | 396 | 408 | 420 | 432 | 444 | 454 | 465 | 475 | 485 | 495 | 506 | 516 | 526 | 537 | 547 | 557 | 567 | 578 | 588 | 598 | 609 |
| 19 | 182 | 195 | 207 | 220 | 232 | 251 | 270 | 290 | 309 | 328 | 336 | 343 | 351 | 358 | 366 | 377 | 389 | 400 | 411 | 423 | 433 | 442 | 452 | 462 | 472 | 482 | 491 | 501 | 511 | 521 | 531 | 540 | 550 | 560 | 570 | 580 |
| 18 | 173 | 185 | 197 | 209 | 220 | 239 | 257 | 275 | 293 | 312 | 319 | 326 | 333 | 341 | 348 | 359 | 369 | 380 | 391 | 402 | 411 | 420 | 430 | 439 | 448 | 458 | 467 | 476 | 485 | 495 | 504 | 513 | 523 | 532 | 541 | 551 |
| 17 | 164 | 175 | 186 | 198 | 209 | 226 | 243 | 261 | 278 | 295 | 302 | 309 | 316 | 323 | 329 | 340 | 350 | 360 | 370 | 381 | 389 | 398 | 407 | 416 | 425 | 433 | 442 | 451 | 460 | 469 | 478 | 486 | 495 | 504 | 513 | 522 |
| 16 | 155 | 165 | 176 | 187 | 197 | 214 | 230 | 246 | 262 | 279 | 285 | 292 | 298 | 305 | 311 | 321 | 330 | 340 | 350 | 359 | 368 | 376 | 384 | 393 | 401 | 409 | 418 | 426 | 434 | 443 | 451 | 459 | 468 | 476 | 484 | 493 |
| 15 | 146 | 156 | 166 | 176 | 186 | 201 | 216 | 232 | 247 | 262 | 268 | 275 | 281 | 287 | 293 | 302 | 311 | 320 | 329 | 338 | 346 | 354 | 362 | 370 | 377 | 385 | 393 | 401 | 409 | 417 | 424 | 432 | 440 | 448 | 456 | 464 |
| 14 | 137 | 146 | 155 | 165 | 174 | 188 | 203 | 217 | 232 | 246 | 252 | 257 | 263 | 269 | 275 | 283 | 292 | 300 | 309 | 317 | 324 | 332 | 339 | 347 | 354 | 361 | 369 | 376 | 383 | 391 | 398 | 405 | 413 | 420 | 427 | 435 |
| 13 | 128 | 136 | 145 | 154 | 162 | 176 | 189 | 203 | 216 | 230 | 235 | 240 | 246 | 251 | 256 | 264 | 272 | 280 | 288 | 296 | 303 | 310 | 317 | 323 | 330 | 337 | 344 | 351 | 358 | 365 | 371 | 378 | 385 | 392 | 399 | 406 |
| 12 | 118 | 127 | 135 | 143 | 151 | 163 | 176 | 188 | 201 | 213 | 218 | 223 | 228 | 233 | 238 | 245 | 253 | 260 | 267 | 275 | 281 | 288 | 294 | 300 | 307 | 313 | 319 | 326 | 332 | 339 | 345 | 351 | 358 | 364 | 370 | 377 |
| 11 | 109 | 117 | 124 | 132 | 139 | 151 | 162 | 174 | 185 | 197 | 201 | 206 | 210 | 215 | 220 | 226 | 233 | 240 | 247 | 254 | 260 | 265 | 271 | 277 | 283 | 289 | 295 | 301 | 307 | 312 | 318 | 324 | 330 | 336 | 342 | 348 |
| 10 | 100 | 107 | 114 | 121 | 128 | 138 | 149 | 159 | 170 | 180 | 185 | 189 | 193 | 197 | 201 | 208 | 214 | 220 | 226 | 233 | 238 | 243 | 249 | 254 | 259 | 265 | 270 | 276 | 281 | 286 | 292 | 297 | 303 | 308 | 313 | 319 |
| 9 | 91 | 97 | 104 | 110 | 116 | 126 | 135 | 145 | 154 | 164 | 168 | 172 | 175 | 179 | 183 | 189 | 194 | 200 | 206 | 211 | 216 | 221 | 226 | 231 | 236 | 241 | 246 | 251 | 256 | 260 | 265 | 270 | 275 | 280 | 285 | 290 |
| 8 | 82 | 88 | 93 | 99 | 104 | 113 | 122 | 130 | 139 | 148 | 151 | 154 | 158 | 161 | 165 | 170 | 175 | 180 | 185 | 190 | 195 | 199 | 203 | 208 | 212 | 217 | 221 | 226 | 230 | 234 | 239 | 243 | 248 | 252 | 256 | 261 |
| 7 | 73 | 78 | 83 | 88 | 93 | 100 | 108 | 116 | 124 | 131 | 134 | 137 | 140 | 143 | 146 | 151 | 156 | 160 | 165 | 169 | 173 | 177 | 181 | 185 | 189 | 193 | 197 | 200 | 204 | 208 | 212 | 216 | 220 | 224 | 228 | 232 |
| 6 | 64 | 68 | 73 | 77 | 81 | 88 | 95 | 101 | 108 | 115 | 117 | 120 | 123 | 125 | 128 | 132 | 136 | 140 | 144 | 148 | 151 | 155 | 158 | 162 | 165 | 169 | 172 | 175 | 179 | 182 | 186 | 189 | 193 | 196 | 199 | 203 |
| 5 | 55 | 58 | 62 | 66 | 70 | 75 | 81 | 87 | 93 | 98 | 101 | 103 | 105 | 108 | 110 | 113 | 117 | 120 | 123 | 127 | 130 | 133 | 136 | 139 | 142 | 144 | 147 | 150 | 153 | 156 | 159 | 162 | 165 | 168 | 171 | 174 |
| 4 | 46 | 49 | 52 | 55 | 58 | 63 | 68 | 72 | 77 | 82 | 84 | 86 | 88 | 90 | 92 | 94 | 97 | 100 | 103 | 106 | 108 | 111 | 113 | 116 | 118 | 120 | 123 | 125 | 128 | 130 | 133 | 135 | 138 | 140 | 142 | 145 |
| 3 | 36 | 39 | 41 | 44 | 46 | 50 | 54 | 58 | 62 | 66 | 67 | 69 | 70 | 72 | 73 | 75 | 78 | 80 | 82 | 85 | 87 | 88 | 90 | 92 | 94 | 96 | 98 | 100 | 102 | 104 | 106 | 108 | 110 | 112 | 114 | 116 |
| 2 | 27 | 29 | 31 | 33 | 35 | 38 | 41 | 43 | 46 | 49 | 50 | 51 | 53 | 54 | 55 | 57 | 58 | 60 | 62 | 63 | 65 | 66 | 68 | 69 | 71 | 72 | 74 | 75 | 77 | 78 | 80 | 81 | 83 | 84 | 85 | 87 |
| 1 | 18 | 19 | 21 | 22 | 23 | 25 | 27 | 29 | 31 | 33 | 34 | 34 | 35 | 36 | 37 | 38 | 39 | 40 | 41 | 42 | 43 | 44 | 45 | 46 | 47 | 48 | 49 | 50 | 51 | 52 | 53 | 54 | 55 | 56 | 57 | 58 |

**Table ST1**: Experimental values for the depth in nanometers for the size and depth sweep shown in Figure 4. These values have been used for the simulations shown in the same figure. The patterning parameters have been adapted as follows: Singly-charged gold ions at a beam current of ~350 pA and a 30 µm beam-forming aperture are used. The step size is 10 nm, circles are exposed in circular outward mode. The base dose value is 1 nC/µm^2^. The dose sweep is linear in 36 steps from a dose factor of 0.1 to 1.85 with constant increment of 0.05. 30 depth have been determined from SEM inspection, the other depth have been extrapolated from these measurements assuming that the depth is linear with dose.

|  | 1 | 2 | 3 | 4 | 5 | 6 | 7 | 8 | 9 | 10 | 11 | 12 | 13 | 14 | 15 | 16 | 17 | 18 | 19 | 20 | 21 | 22 | 23 | 24 | 25 | 26 | 27 | 28 | 29 | 30 | 31 | 32 | 33 | 34 | 35 | 36 |
| --- | --- | --- | --- | --- | --- | --- | --- | --- | --- | --- | --- | --- | --- | --- | --- | --- | --- | --- | --- | --- | --- | --- | --- | --- | --- | --- | --- | --- | --- | --- | --- | --- | --- | --- | --- | --- |
| 36 | 400 | 410 | 420 | 430 | 440 | 450 | 460 | 470 | 480 | 490 | 500 | 510 | 520 | 530 | 540 | 550 | 560 | 570 | 580 | 590 | 600 | 610 | 620 | 630 | 640 | 650 | 660 | 670 | 680 | 690 | 700 | 710 | 720 | 730 | 740 | 750 |
| 35 | 399 | 409 | 419 | 429 | 439 | 449 | 459 | 469 | 479 | 489 | 499 | 509 | 519 | 529 | 539 | 549 | 559 | 569 | 579 | 589 | 599 | 609 | 619 | 629 | 639 | 649 | 659 | 669 | 679 | 689 | 699 | 709 | 719 | 729 | 739 | 749 |
| 34 | 398 | 408 | 418 | 428 | 438 | 448 | 458 | 468 | 478 | 488 | 498 | 508 | 518 | 528 | 538 | 548 | 558 | 568 | 578 | 588 | 598 | 608 | 618 | 628 | 638 | 648 | 658 | 668 | 678 | 688 | 698 | 708 | 718 | 728 | 738 | 748 |
| 33 | 396 | 406 | 416 | 426 | 436 | 446 | 456 | 466 | 476 | 486 | 496 | 506 | 516 | 526 | 536 | 546 | 556 | 566 | 576 | 586 | 596 | 606 | 616 | 626 | 636 | 646 | 656 | 666 | 676 | 686 | 696 | 706 | 716 | 726 | 736 | 746 |
| 32 | 395 | 405 | 415 | 425 | 435 | 445 | 455 | 465 | 475 | 485 | 495 | 505 | 515 | 525 | 535 | 545 | 555 | 565 | 575 | 585 | 595 | 605 | 615 | 625 | 635 | 645 | 655 | 665 | 675 | 685 | 695 | 705 | 715 | 725 | 735 | 745 |
| 31 | 394 | 404 | 414 | 424 | 434 | 444 | 454 | 464 | 474 | 484 | 494 | 504 | 514 | 524 | 534 | 544 | 554 | 564 | 574 | 584 | 594 | 604 | 614 | 624 | 634 | 644 | 654 | 664 | 674 | 684 | 694 | 704 | 714 | 724 | 734 | 744 |
| 30 | 393 | 403 | 413 | 423 | 433 | 443 | 453 | 463 | 473 | 483 | 493 | 503 | 513 | 523 | 533 | 543 | 553 | 563 | 573 | 583 | 593 | 603 | 613 | 623 | 633 | 643 | 653 | 663 | 673 | 683 | 693 | 703 | 713 | 723 | 733 | 743 |
| 29 | 392 | 402 | 412 | 422 | 432 | 442 | 452 | 462 | 472 | 482 | 492 | 502 | 512 | 522 | 532 | 542 | 552 | 562 | 572 | 582 | 592 | 602 | 612 | 622 | 632 | 642 | 652 | 662 | 672 | 682 | 692 | 702 | 712 | 722 | 732 | 742 |
| 28 | 391 | 401 | 411 | 421 | 431 | 441 | 451 | 461 | 471 | 481 | 491 | 501 | 511 | 521 | 531 | 541 | 551 | 561 | 571 | 581 | 591 | 601 | 611 | 621 | 631 | 641 | 651 | 661 | 671 | 681 | 691 | 701 | 711 | 721 | 731 | 741 |
| 27 | 389 | 399 | 409 | 419 | 429 | 439 | 449 | 459 | 469 | 479 | 489 | 499 | 509 | 519 | 529 | 539 | 549 | 559 | 569 | 579 | 589 | 599 | 609 | 619 | 629 | 639 | 649 | 659 | 669 | 679 | 689 | 699 | 709 | 719 | 729 | 739 |
| 26 | 388 | 398 | 408 | 418 | 428 | 438 | 448 | 458 | 468 | 478 | 488 | 498 | 508 | 518 | 528 | 538 | 548 | 558 | 568 | 578 | 588 | 598 | 608 | 618 | 628 | 638 | 648 | 658 | 668 | 678 | 688 | 698 | 708 | 718 | 728 | 738 |
| 25 | 387 | 397 | 407 | 417 | 427 | 437 | 447 | 457 | 467 | 477 | 487 | 497 | 507 | 517 | 527 | 537 | 547 | 557 | 567 | 577 | 587 | 597 | 607 | 617 | 627 | 637 | 647 | 657 | 667 | 677 | 687 | 697 | 707 | 717 | 727 | 737 |
| 24 | 386 | 396 | 406 | 416 | 426 | 436 | 446 | 456 | 466 | 476 | 486 | 496 | 506 | 516 | 526 | 536 | 546 | 556 | 566 | 576 | 586 | 596 | 606 | 616 | 626 | 636 | 646 | 656 | 666 | 676 | 686 | 696 | 706 | 716 | 726 | 736 |
| 23 | 385 | 395 | 405 | 415 | 425 | 435 | 445 | 455 | 465 | 475 | 485 | 495 | 505 | 515 | 525 | 535 | 545 | 555 | 565 | 575 | 585 | 595 | 605 | 615 | 625 | 635 | 645 | 655 | 665 | 675 | 685 | 695 | 705 | 715 | 725 | 735 |
| 22 | 384 | 394 | 404 | 414 | 424 | 434 | 444 | 454 | 464 | 474 | 484 | 494 | 504 | 514 | 524 | 534 | 544 | 554 | 564 | 574 | 584 | 594 | 604 | 614 | 624 | 634 | 644 | 654 | 664 | 674 | 684 | 694 | 704 | 714 | 724 | 734 |
| 21 | 381 | 391 | 401 | 411 | 421 | 431 | 441 | 451 | 461 | 471 | 481 | 491 | 501 | 511 | 521 | 531 | 541 | 551 | 561 | 571 | 581 | 591 | 601 | 611 | 621 | 631 | 641 | 651 | 661 | 671 | 681 | 691 | 701 | 711 | 721 | 731 |
| 20 | 382 | 392 | 402 | 412 | 422 | 432 | 442 | 452 | 462 | 472 | 482 | 492 | 502 | 512 | 522 | 532 | 542 | 552 | 562 | 572 | 582 | 592 | 602 | 612 | 622 | 632 | 642 | 652 | 662 | 672 | 682 | 692 | 702 | 712 | 722 | 732 |
| 19 | 381 | 391 | 401 | 411 | 421 | 431 | 441 | 451 | 461 | 471 | 481 | 491 | 501 | 511 | 521 | 531 | 541 | 551 | 561 | 571 | 581 | 591 | 601 | 611 | 621 | 631 | 641 | 651 | 661 | 671 | 681 | 691 | 701 | 711 | 721 | 731 |
| 18 | 380 | 390 | 400 | 410 | 420 | 430 | 440 | 450 | 460 | 470 | 480 | 490 | 500 | 510 | 520 | 530 | 540 | 550 | 560 | 570 | 580 | 590 | 600 | 610 | 620 | 630 | 640 | 650 | 660 | 670 | 680 | 690 | 700 | 710 | 720 | 730 |
| 17 | 377 | 387 | 397 | 407 | 417 | 427 | 437 | 447 | 457 | 467 | 477 | 487 | 497 | 507 | 517 | 527 | 537 | 547 | 557 | 567 | 577 | 587 | 597 | 607 | 617 | 627 | 637 | 647 | 657 | 667 | 677 | 687 | 697 | 707 | 717 | 727 |
| 16 | 374 | 384 | 394 | 404 | 414 | 424 | 434 | 444 | 454 | 464 | 474 | 484 | 494 | 504 | 514 | 524 | 534 | 544 | 554 | 564 | 574 | 584 | 594 | 604 | 614 | 624 | 634 | 644 | 654 | 664 | 674 | 684 | 694 | 704 | 714 | 724 |
| 15 | 371 | 381 | 391 | 401 | 411 | 421 | 431 | 441 | 451 | 461 | 471 | 481 | 491 | 501 | 511 | 521 | 531 | 541 | 551 | 561 | 571 | 581 | 591 | 601 | 611 | 621 | 631 | 641 | 651 | 661 | 671 | 681 | 691 | 701 | 711 | 721 |
| 14 | 368 | 378 | 388 | 398 | 408 | 418 | 428 | 438 | 448 | 458 | 468 | 478 | 488 | 498 | 508 | 518 | 528 | 538 | 548 | 558 | 568 | 578 | 588 | 598 | 608 | 618 | 628 | 638 | 648 | 658 | 668 | 678 | 688 | 698 | 708 | 718 |
| 13 | 365 | 375 | 385 | 395 | 405 | 415 | 425 | 435 | 445 | 455 | 465 | 475 | 485 | 495 | 505 | 515 | 525 | 535 | 545 | 555 | 565 | 575 | 585 | 595 | 605 | 615 | 625 | 635 | 645 | 655 | 665 | 675 | 685 | 695 | 705 | 715 |
| 12 | 362 | 372 | 382 | 392 | 402 | 412 | 422 | 432 | 442 | 452 | 462 | 472 | 482 | 492 | 502 | 512 | 522 | 532 | 542 | 552 | 562 | 572 | 582 | 592 | 602 | 612 | 622 | 632 | 642 | 652 | 662 | 672 | 682 | 692 | 702 | 712 |
| 11 | 359 | 369 | 379 | 389 | 399 | 409 | 419 | 429 | 439 | 449 | 459 | 469 | 479 | 489 | 499 | 509 | 519 | 529 | 539 | 549 | 559 | 569 | 579 | 589 | 599 | 609 | 619 | 629 | 639 | 649 | 659 | 669 | 679 | 689 | 699 | 709 |
| 10 | 356 | 366 | 376 | 386 | 396 | 406 | 416 | 426 | 436 | 446 | 456 | 466 | 476 | 486 | 496 | 506 | 516 | 526 | 536 | 546 | 556 | 566 | 576 | 586 | 596 | 606 | 616 | 626 | 636 | 646 | 656 | 666 | 676 | 686 | 696 | 706 |
| 9 | 354 | 364 | 374 | 384 | 394 | 404 | 414 | 424 | 434 | 444 | 454 | 464 | 474 | 484 | 494 | 504 | 514 | 524 | 534 | 544 | 554 | 564 | 574 | 584 | 594 | 604 | 614 | 624 | 634 | 644 | 654 | 664 | 674 | 684 | 694 | 704 |
| 8 | 351 | 361 | 371 | 381 | 391 | 401 | 411 | 421 | 431 | 441 | 451 | 461 | 471 | 481 | 491 | 501 | 511 | 521 | 531 | 541 | 551 | 561 | 571 | 581 | 591 | 601 | 611 | 621 | 631 | 641 | 651 | 661 | 671 | 681 | 691 | 701 |
| 7 | 348 | 358 | 368 | 378 | 388 | 398 | 408 | 418 | 428 | 438 | 448 | 458 | 468 | 478 | 488 | 498 | 508 | 518 | 528 | 538 | 548 | 558 | 568 | 578 | 588 | 598 | 608 | 618 | 628 | 638 | 648 | 658 | 668 | 678 | 688 | 698 |
| 6 | 345 | 355 | 365 | 375 | 385 | 395 | 405 | 415 | 425 | 435 | 445 | 455 | 465 | 475 | 485 | 495 | 505 | 515 | 525 | 535 | 545 | 555 | 565 | 575 | 585 | 595 | 605 | 615 | 625 | 635 | 645 | 655 | 665 | 675 | 685 | 695 |
| 5 | 342 | 352 | 362 | 372 | 382 | 392 | 402 | 412 | 422 | 432 | 442 | 452 | 462 | 472 | 482 | 492 | 502 | 512 | 522 | 532 | 542 | 552 | 562 | 572 | 582 | 592 | 602 | 612 | 622 | 632 | 642 | 652 | 662 | 672 | 682 | 692 |
| 4 | 339 | 349 | 359 | 369 | 379 | 389 | 399 | 409 | 419 | 429 | 439 | 449 | 459 | 469 | 479 | 489 | 499 | 509 | 519 | 529 | 539 | 549 | 559 | 569 | 579 | 589 | 599 | 609 | 619 | 629 | 639 | 649 | 659 | 669 | 679 | 689 |
| 3 | 336 | 346 | 356 | 366 | 376 | 386 | 396 | 406 | 416 | 426 | 436 | 446 | 456 | 466 | 476 | 486 | 496 | 506 | 516 | 526 | 536 | 546 | 556 | 566 | 576 | 586 | 596 | 606 | 616 | 626 | 636 | 646 | 656 | 666 | 676 | 686 |
| 2 | 333 | 343 | 353 | 363 | 373 | 383 | 393 | 403 | 413 | 423 | 433 | 443 | 453 | 463 | 473 | 483 | 493 | 503 | 513 | 523 | 533 | 543 | 553 | 563 | 573 | 583 | 593 | 603 | 613 | 623 | 633 | 643 | 653 | 663 | 673 | 683 |
| 1 | 330 | 340 | 350 | 360 | 370 | 380 | 390 | 400 | 410 | 420 | 430 | 440 | 450 | 460 | 470 | 480 | 490 | 500 | 510 | 520 | 530 | 540 | 550 | 560 | 570 | 580 | 590 | 600 | 610 | 620 | 630 | 640 | 650 | 660 | 670 | 680 |

**Table ST2**: Experimental values for the diameter in nanometers for the size and depth sweep shown in Figure 4. These values have been used for the simulations shown in the same figure. The design parameters in the GDSII file are diameters from 150 nm to 500 nm in steps of 10 nm. 30 diameters have been determined from SEM inspection, the other have been extrapolated from these measurements assuming that the diameters increases linearly with the depth (or dose).


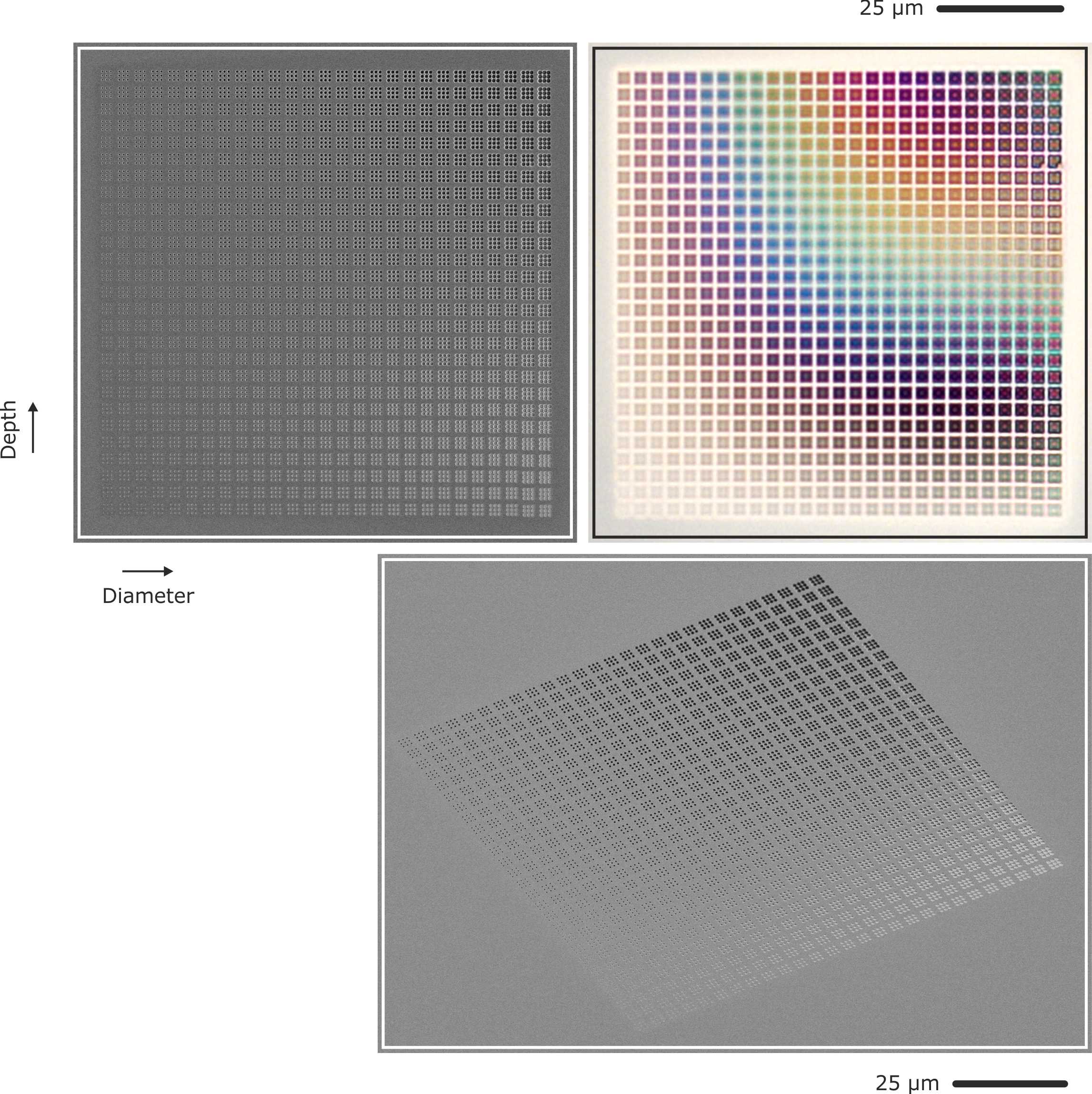


**Figure S9**: SEM and optical microscope images of a diameter and depth sweep. The normal and tilted view SEM images show the increasing hole diameter in horizontal and the increasing depth in vertical direction. Periodicity is fixed at 900nm in both directions. Each variation contains 3x3 holes, the spacing is 3600 nm. The optical microscope image illustrates the distinct colour appearance of the individual 3x3 arrays as well as the excellent tuneability of the colour. Please note that apart from “colours” also whitish and blackish appearance can be created.


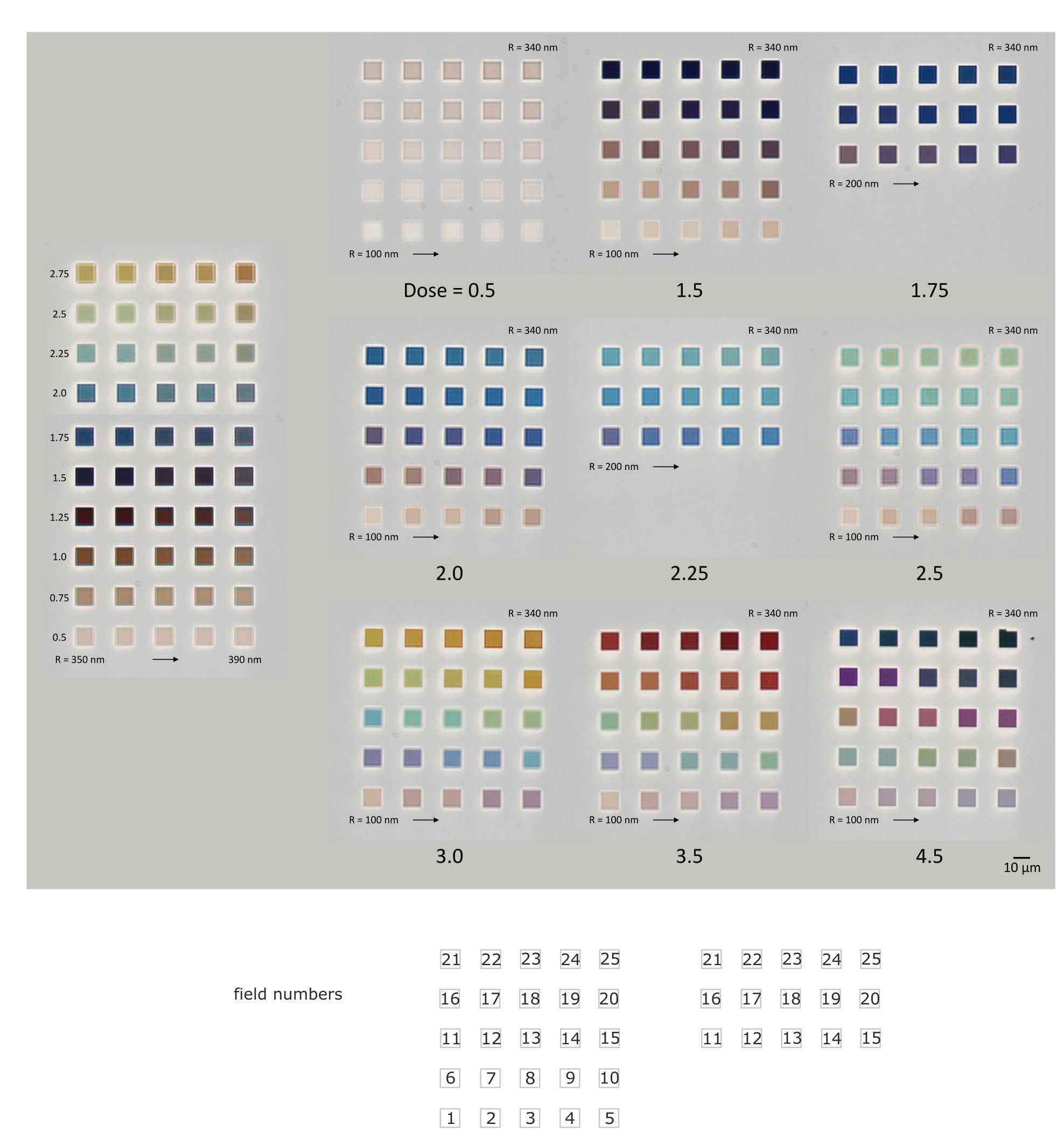


**Figure S10**: Colour catalogue: Composed optical microscope image of the parameter space in use. All parameters given in the figure are the design values used for the structuring. The resulting experimentally obtained parameters for diameter and depth can be found in the Table ST3.


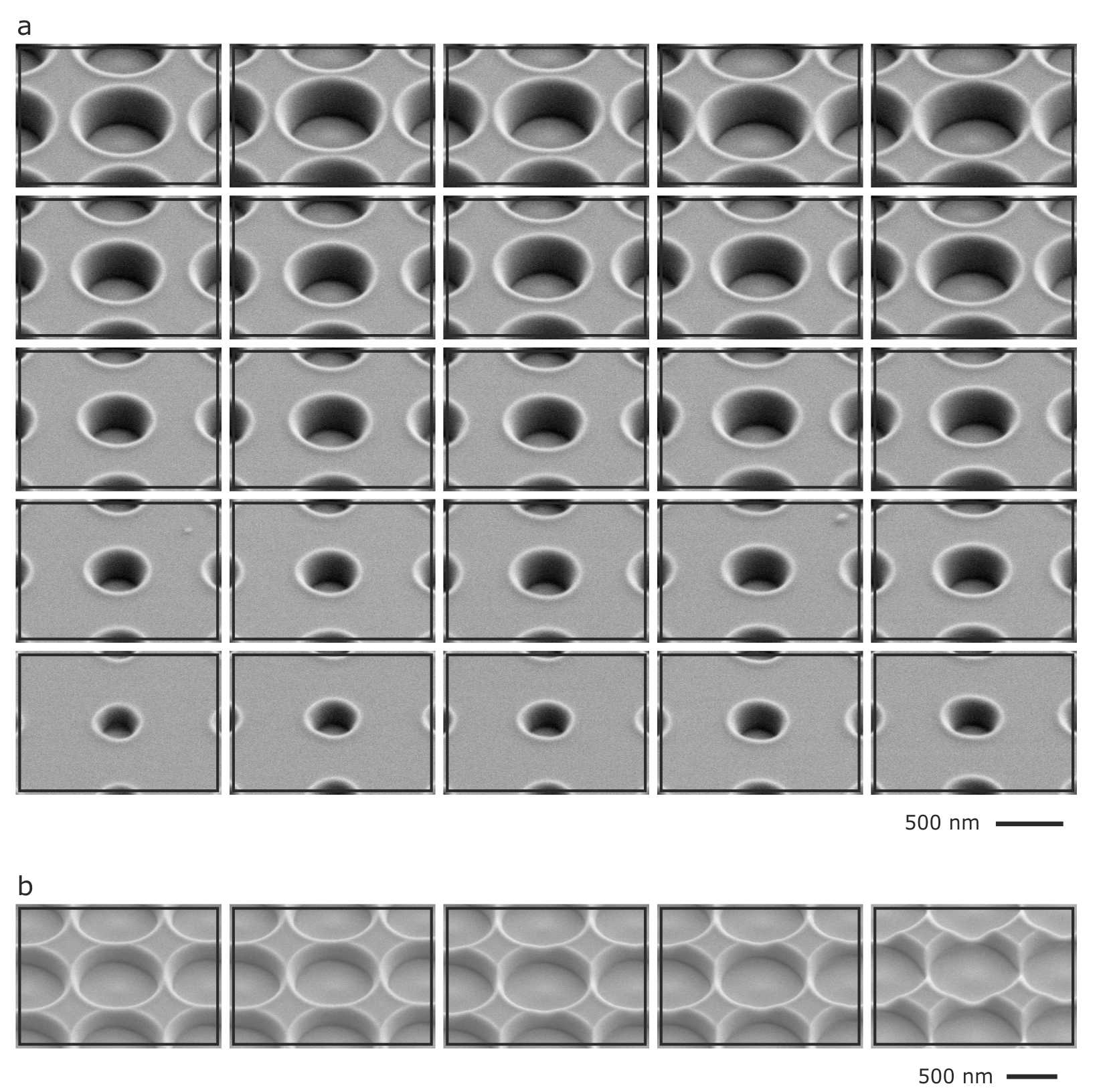


**Figure S11**: SEM images illustrating the size variation of the colour catalogue shown in Figure S10. Panel **a** depicts the sizes of the 5x5 arrays (full and reduced one), panel **b** depicts the 5x10 array on the left side of Figure S10. Exact parameters are given in Table ST3.


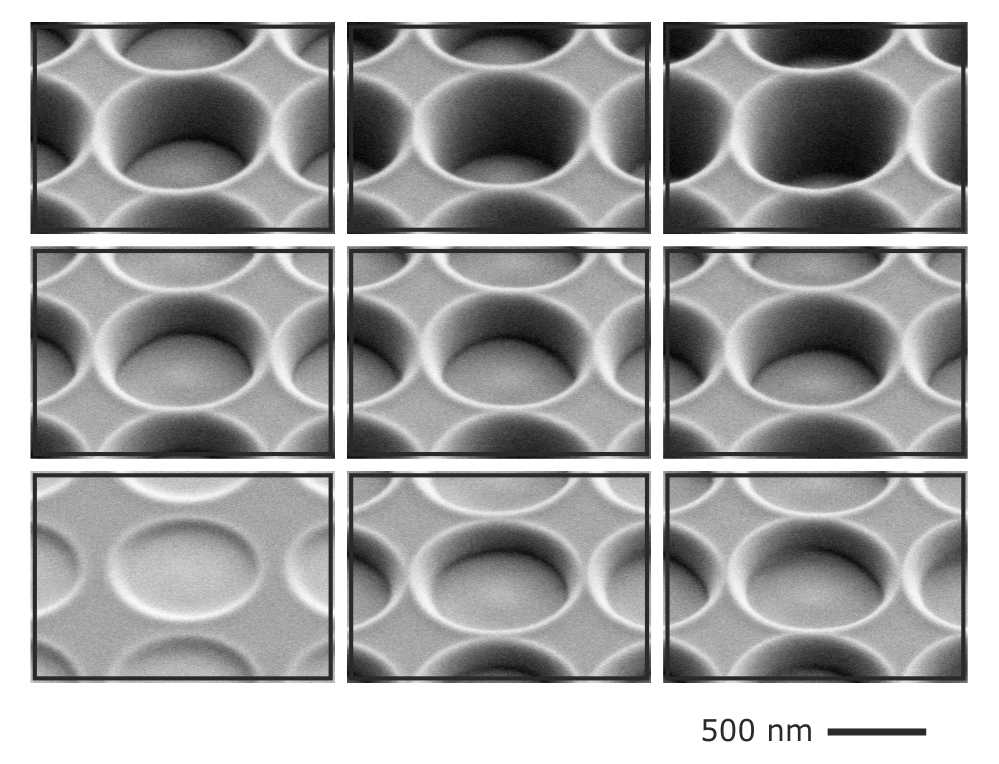


**Figure S12**: SEM images illustrating the depth variation of the colour catalogue shown in Figure S10. The resulting depth depends mainly on the deposited dose, that is, the number of ions. However, there is also slight dependence on the diameter, larger circles tend to be slightly deeper at the same dose value as smaller ones. For intermediate diameters around 600 nm the dose values result in depth as follows: Dose 0.5: 60 nm; Dose 1.50: 170 nm; Dose 1.75: 200 nm; Dose 2.25: 260 nm; Dose 2.50: 290 nm; Dose 3.00: 345 nm; 3.50: 400 nm; Dose 4.50: 515 nm. For the size sweep we observe depth within +/- 10% around these values.


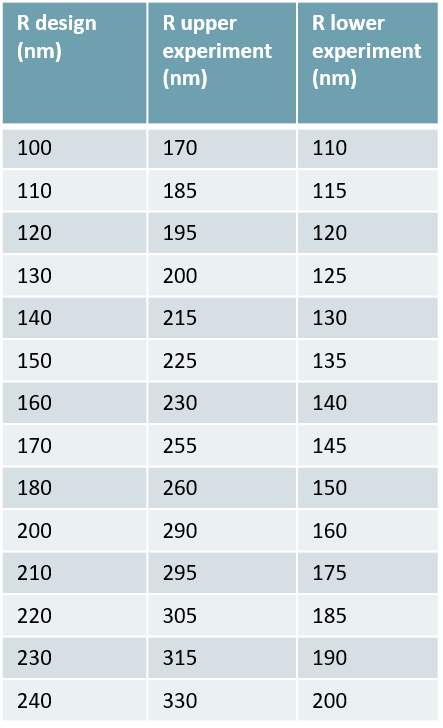

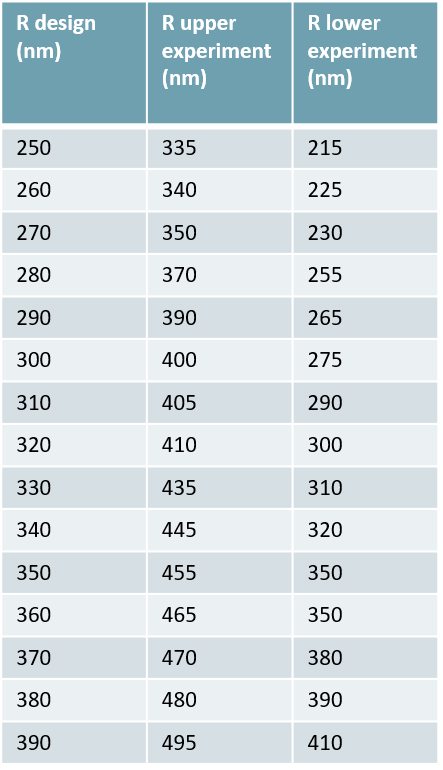


**Table ST3**: Experimental values for the upper and lower diameter of the cylindrical voids, nomenclature as in Figure S10.


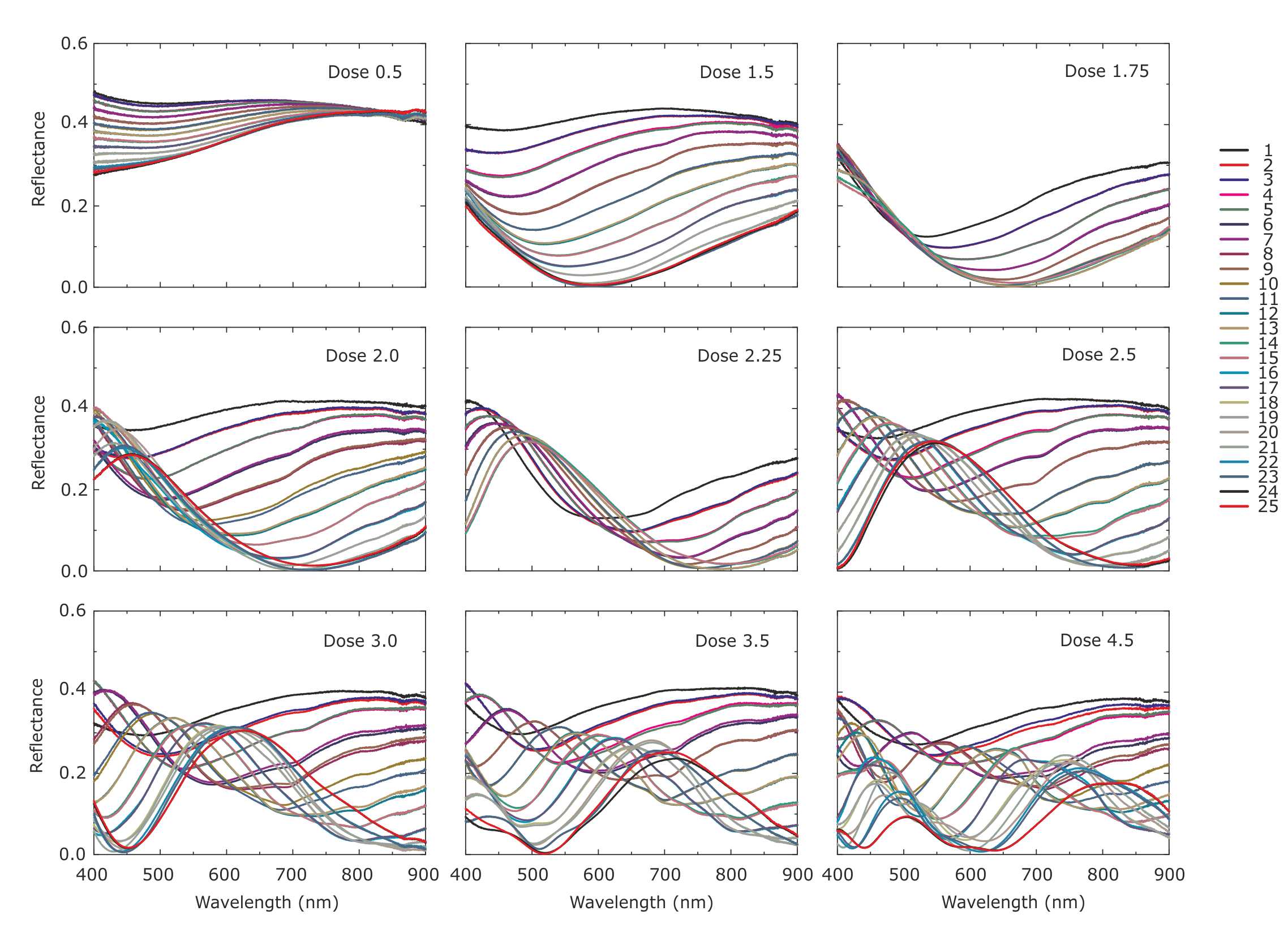


**Figure S13**: Unpolarised reflection spectra of the structures shown in Figure S10 with the parameter given in Table ST3. Please note, that except for dose values 1.75 and 2.25, all graphs contain 25 spectra corresponding to the 25 different diameters. For dose factors 1.75 and 2.25 the 15 largest diameters have been structured. For the experimental dimensions and the arrangement please refer to Figure S10 and table ST3. Please also note that two structures each are very similar in the experimentally realized dimensions and the spectra are thus also very similar and do partially overlap.


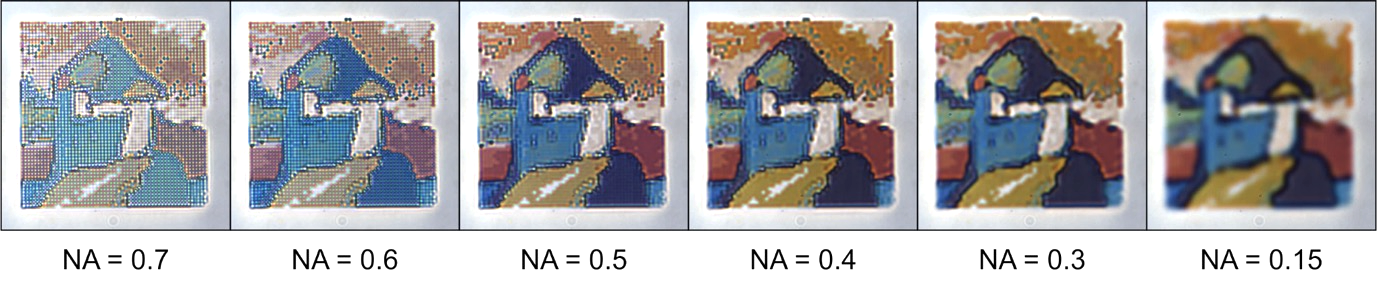


**Figure S14: Images of a section of the colour printed “Improvisation No. 9” for varying numerical aperture (NA) of the microscope objective.** The most straightforward and intuitive experimental method to study the angle dependence is imaging based: Utilizing a k-space microscopy setup, we can vary the NA with which we image a structure. A larger NA implies a larger spread of incident and out going angles (which will also result in a better spatial resolution). We imaged the section of the colour printed version of “Improvisation No. 9” shown in Figure 5 of the main manuscript with varying NA. One can observe two main feature: The colour impression changes only slightly, underpinning the resonant localized nature of the modes. Additionally, one can clearly observe a larger spatial resolution of the image, which is the main reason for the slight fading of the colour.

λ (nm) n k

230 1.52474 3.62384

232 1.64355 3.49551

234 1.69363 3.43674

236 1.70027 3.4252

238 1.68955 3.43865

240 1.68209 3.45175

242 1.67916 3.46

244 1.68311 3.51011

246 1.68258 3.56351

248 1.68432 3.62043

250 1.69492 3.6803

252 1.71159 3.74609

254 1.73575 3.82204

256 1.74741 3.90751

258 1.77825 3.99607

260 1.80576 4.09502

262 1.8474 4.20295

264 1.90089 4.31412

266 1.96679 4.44308

268 2.04371 4.58143

270 2.15406 4.72433

272 2.29679 4.86819

274 2.46614 4.99711

276 2.65159 5.1122

278 2.87105 5.20665

280 3.09979 5.27456

282 3.33034 5.32035

284 3.60146 5.35364

286 3.89557 5.35113

288 4.20301 5.28144

290 4.49059 5.12703

292 4.72925 4.91355

294 4.88765 4.68184

296 4.98159 4.46423

298 5.03318 4.26642

300 5.06448 4.09359

302 5.07841 3.94523

304 5.07994 3.82129

306 5.07719 3.71398

308 5.07367 3.621

310 5.07674 3.53956

312 5.07416 3.46823

314 5.08066 3.40653

316 5.08703 3.35155

318 5.09131 3.30462

320 5.10631 3.25722

322 5.11214 3.2194

324 5.12355 3.18084

326 5.14194 3.14341

328 5.16594 3.10927

330 5.18356 3.07678

332 5.19874 3.04667

334 5.22578 3.01594

336 5.25029 2.99213

338 5.2743 2.96867

340 5.30124 2.94738

342 5.33389 2.93184

344 5.36549 2.91917

346 5.4015 2.91026

348 5.44479 2.90417

350 5.49781 2.90401

352 5.56231 2.90892

354 5.64408 2.91877

356 5.75222 2.9246

358 5.886 2.92787

360 6.05492 2.90309

362 6.25366 2.82993

364 6.46277 2.70096

366 6.65189 2.51186

368 6.79771 2.26735

370 6.88427 1.99102

372 6.90837 1.70132

374 6.8659 1.43182

376 6.77072 1.19176

λ (nm) n k

378 6.65163 0.99827

380 6.5202 0.84581

382 6.37989 0.71943

384 6.25958 0.62819

386 6.15173 0.55649

388 6.04901 0.4978

390 5.95257 0.44777

392 5.86415 0.40687

394 5.7879 0.37281

396 5.71087 0.34173

398 5.63775 0.31526

400 5.57228 0.29177

402 5.50921 0.27116

404 5.4497 0.25434

406 5.39534 0.23907

408 5.34126 0.22436

410 5.29125 0.21202

412 5.2446 0.20015

414 5.20121 0.18973

416 5.15893 0.18035

418 5.11706 0.17127

420 5.08015 0.16301

422 5.04452 0.15631

424 5.00875 0.14914

426 4.97646 0.14318

428 4.9444 0.13711

430 4.91572 0.13137

432 4.88744 0.12617

434 4.86016 0.12158

436 4.83602 0.11709

438 4.81035 0.11366

440 4.78542 0.10863

442 4.76109 0.10565

444 4.73756 0.10179

446 4.71354 0.09847

448 4.69082 0.09521

450 4.66869 0.0912

452 4.64783 0.08856

454 4.62756 0.08587

456 4.60791 0.08338

458 4.58882 0.08106

460 4.57017 0.07889

462 4.5518 0.07687

464 4.53401 0.07483

466 4.5171 0.07242

468 4.50091 0.06989

470 4.48517 0.06764

472 4.4697 0.06604

474 4.45451 0.06462

476 4.43941 0.06307

478 4.42453 0.06146

480 4.4101 0.05991

482 4.39632 0.05849

484 4.38282 0.05711

486 4.36911 0.05568

488 4.35554 0.05423

490 4.34269 0.05284

492 4.33105 0.05156

494 4.31996 0.05035

496 4.30906 0.04918

498 4.29825 0.04806

500 4.28735 0.04701

502 4.27614 0.04606

504 4.26513 0.0451

506 4.25485 0.04395

508 4.24511 0.04272

510 4.23558 0.04162

512 4.22603 0.04083

514 4.21654 0.04011

516 4.20697 0.03935

518 4.19746 0.03855

520 4.1882 0.03773

522 4.17938 0.03691

524 4.17082 0.03612

λ (nm) n k

526 4.16244 0.03545

528 4.15426 0.03486

530 4.1464 0.03429

532 4.13902 0.03373

534 4.13158 0.03311

536 4.12329 0.03223

538 4.11469 0.03127

540 4.10671 0.03052

542 4.10013 0.03024

544 4.09394 0.03005

546 4.08745 0.02973

548 4.08076 0.02929

550 4.07408 0.0288

552 4.06752 0.02825

554 4.06097 0.02768

556 4.05433 0.02709

558 4.04767 0.02649

560 4.04108 0.02586

562 4.03461 0.02517

564 4.02839 0.02462

566 4.02283 0.02453

568 4.01761 0.02465

570 4.01224 0.02462

572 4.00628 0.02408

574 4.00022 0.02347

576 3.99426 0.0231

578 3.98848 0.02288

580 3.98293 0.02261

582 3.97772 0.02217

584 3.97275 0.02179

586 3.96801 0.02175

588 3.96342 0.02187

590 3.95888 0.02183

592 3.95428 0.02137

594 3.94968 0.02079

596 3.94507 0.02019

598 3.94047 0.01965

600 3.93589 0.01926

602 3.93134 0.01913

604 3.92684 0.0191

606 3.92239 0.01911

608 3.91799 0.01909

610 3.91362 0.01893

612 3.90927 0.01851

614 3.90497 0.01804

616 3.90075 0.01768

618 3.89664 0.01741

620 3.89276 0.01715

622 3.88921 0.01687

624 3.8858 0.01659

626 3.88243 0.01624

628 3.8791 0.01591

630 3.8759 0.0157

632 3.87293 0.01574

634 3.8698 0.0158

636 3.866 0.01563

638 3.86183 0.01533

640 3.85774 0.01508

642 3.85408 0.015

644 3.85067 0.01493

646 3.84764 0.01472

648 3.8448 0.01442

650 3.84184 0.01417

652 3.83846 0.01403

654 3.83515 0.01393

656 3.83244 0.01384

658 3.82999 0.01373

660 3.82719 0.01354

662 3.82349 0.01319

664 3.81983 0.01288

666 3.8172 0.01282

668 3.81506 0.01289

670 3.81249 0.01282

672 3.80867 0.01238

λ (nm) n k

674 3.8048 0.01198

676 3.80183 0.0121

678 3.79947 0.01247

680 3.7972 0.01264

682 3.79469 0.01222

684 3.79216 0.01173

686 3.78967 0.0116

688 3.7872 0.01164

690 3.7847 0.01149

692 3.78214 0.01083

694 3.77951 0.01023

696 3.77666 0.01022

698 3.77378 0.01058

700 3.77117 0.01094

702 3.76913 0.01105

704 3.76713 0.01103

706 3.76455 0.01083

708 3.76176 0.0105

710 3.75938 0.01012

712 3.75799 0.00975

714 3.75665 0.00946

716 3.75456 0.00943

718 3.75196 0.00957

720 3.74931 0.00969

722 3.74687 0.00964

724 3.7445 0.00966

726 3.74205 0.01017

728 3.73975 0.01077

730 3.73801 0.01083

732 3.73728 0.00971

734 3.73658 0.0085

736 3.73493 0.00822

738 3.73265 0.00852

740 3.73027 0.00879

742 3.72816 0.00863

744 3.72604 0.00832

746 3.72362 0.00777

748 3.72125 0.0072

750 3.71959 0.00694

752 3.71931 0.00734

754 3.719 0.00785

756 3.71731 0.00814

758 3.71466 0.00819

760 3.71168 0.00804

762 3.70867 0.00767

764 3.70618 0.00717

766 3.70556 0.0065

768 3.70583 0.00589

770 3.70539 0.00568

772 3.70282 0.00627

774 3.69987 0.00694

776 3.69763 0.00704

778 3.69593 0.00675

780 3.6944 0.0063

782 3.69291 0.00579

784 3.69138 0.00545

786 3.68954 0.00562

788 3.68759 0.00619

790 3.68577 0.00703

792 3.68431 0.00814

794 3.68317 0.0089

796 3.68278 0.00832

798 3.68262 0.00691

800 3.68182 0.00548

802 3.67956 0.00461

804 3.6771 0.00421

806 3.6755 0.00481

808 3.6744 0.00594

810 3.67314 0.00688

812 3.67123 0.00704

814 3.66933 0.00683

816 3.66799 0.00615

818 3.66696 0.00528

820 3.66573 0.00468

λ (nm) n k

822 3.66386 0.00474

824 3.66227 0.005

826 3.6622 0.00526

828 3.66276 0.00543

830 3.66229 0.00548

832 3.65927 0.0053

834 3.65637 0.005

836 3.65476 0.00464

838 3.65559 0.00423

840 3.66003 0.00382

Table ST4: Dielectric data used in Figure 2 in the main text. Taken from http://www.sspectra.com/sopra.html
